# Unprecedented efficient electron transport across Au nanoparticles with up to 25-nm insulating SiO_2_-shells

**DOI:** 10.1038/s41598-019-54835-2

**Published:** 2019-12-04

**Authors:** Chuanping Li, Chen Xu, David Cahen, Yongdong Jin

**Affiliations:** 1 State Key Laboratory of Electroanalytical Chemistry, Changchun Institute of Applied Chemistry, Chinese Academy of Sciences, 5625 Renmin Street, Changchun, 130022 P.R. China; 2 University of Chinese Academy of Sciences, Beijing, 100049 P.R. China; 3 University of Science and Technology of China, Hefei, Anhui 230029 P.R. China; 4 Department of Materials and Interfaces, Weizmann Institute of Science, Rehovot, 76100 Israel

**Keywords:** Chemistry, Materials science, Nanoscience and technology, Physics

## Abstract

Quantum tunneling is the basis of molecular electronics, but often its electron transport range is too short to overcome technical defects caused by downscaling of electronic devices, which limits the development of molecular-/nano-electronics. Marrying electronics with plasmonics may well present a revolutionary way to meet this challenge as it can manipulate electron flow with plasmonics at the nanoscale. Here we report on unusually efficient temperature-independent electron transport, with some photoconductivity, across a new type of junction with active plasmonics. The junction is made by assembly of SiO_2_ shell-insulated Au nanoparticles (Au@SiO_2_ NPs) into dense nanomembranes of a few Au@SiO_2_ layers thick and transport is measured across these membranes. We propose that the mechanism is plasmon-enabled transport, possibly tunneling (as it is temperature-independent). Unprecedentedly ultra-long-range transport across one, up to even three layers of Au@SiO_2_ in the junction, with a cumulative insulating (silica) gap up to 29 nm/NP layer was achieved, well beyond the measurable limit for normal quantum mechanical tunneling across insulators (~2.5 nm at 0.5–1 V). This finding opens up a new interdisciplinary field of exploration in nanoelectronics with wide potential impact on such areas as electronic information transfer.

## Introduction

Stimulated by the seminal use of functional molecules as building blocks for electronic devices^1–3^, researchers all over the world have been pursuing molecular-/nano-electronics and exploring electron transport phenomena thereof for a few decades^1,2,4–9^. Up to now, researches have been reported to explore the electron transport behavior (eg. single electron tunneling, SET; resonant tunneling) by using scanning tunneling microscope (STM) or atomic force microscope (AFM)^10–12^. Although significant progress was made in the field, there is still a long way to success for practical use, primarily due to the long-standing technical barriers^13,14^ in fabricating complete robust and reliable electronic nanodevices (like junctions and transistors) with nm-scale precision and high yields. Meanwhile, due to the distance limitation of efficient quantum tunneling, the electron transport distance in such devices is too short (e.g., the distance across which tunneling through organic alkane (conjugated) molecules can be measured is <4.5 nm)2 to bypass short-channel effects (in Si transistors) or circumvent short-circuits due to the downscaling of electronic elements^15^. This dilemma seriously hinders development and practical applications of molecular and, in general nm-scale electronics.

**Figure 1:**
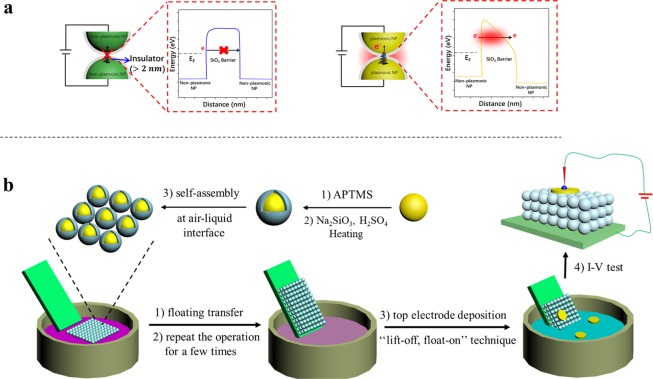
Electron transport regimes (across an insulating gap) and the procedures for preparing sandwich-type meta-junction. Schematic illustrations of (**a**) the accepted quantum tunneling (left) and the suggested plasmon-enabled long-range electron tunneling (P-transport) regimes (right). (**b**) All-solution process to prepare the sandwich-type Au@SiO_2_ nanomembrane meta-junctions.

Plasmonics, which converts light into propagating electrical signals via excitation of surface plasmons, merges photonics with electronics at the nanoscale to circumvent the diffraction limit^1,2^ and, therefore, provides a possible means to manipulate electron flow with it and a revolutionary path to next-generation electronics^16^. It is now well known that the surrounding environment, size and shape of the nanoparticles play great roles on the optical and electrical plasmonic properities^17–19^. However, little is known so far about electron transport *cum* plasmonics on the nanoscale, due to extreme technical difficulties to realize suitable current-carrying nanodevices that can be studied controllably and systematically. Electron transport on plasmonic AuNPs may behave different (than their bulk counterparts) as such NPs can be regarded as the small, nanoscale electron accelerators, i.e., they accelerate nearby moving electrons due to the presence of (inherent) collective high-energy electron oscillations (plasmons) and the strong electromagnetic field on AuNP surfaces. As we will show here, and as schematically illustrated in a cartoon (Fig. 1a, right), incorporating AuNPs into an insulator junction, can allow measurable electron transport across an insulating gap that can be up to an order of magnitude wider than what can be expected for “normal” tunneling across insulators.

Herein we finely prepared a monolayered 2D electronic metamaterial, in this case from 1 to 3 Au@SiO_2_ NP layers thick, at top and bottom, to form a planar junction, the length of which is determined by the thickness of the membrane. We call such devices *meta-junctions* since as we will show, transport across the resulting sandwich-type junction is affected by active plasmonics. The insulating SiO_2_ shell not only presents a barrier for direct conduction from Au to Au NP, it also prevents possible electromigration of Au (to form filaments) during electrical measurements. Thus, it creates a nanoscale transport barrier that is adjustable by varying shell thickness. Here the shell-insulated AuNPs act as both electron transfer mediators and plasmonically active elements. Figure 1b shows the schematic of the all-solution process to prepare the meta-junctions. By varying the silica-shell thickness and the number of layers in the stacked few-layer junction architecture, the platform allows us for systematic probing of long-range electron tunneling and possible scaling behavior. More importantly, the rigid structure of the silica shells, the compactness of the nanomembrane, along with the “soft” top electrode deposition by the left off-float on (LOFO) technique^20^, all of which minimize the chance for short-circuits, allow for reliable and reproducible current-voltage (I-V) measurements of the resulting junctions. We find long-range electron transport (ET) not only across a monolayer, but even a trilayer junction of Au@SiO_2_ NPs, with a cumulative insulating (silica) gap up to 29 nm/NP layer, which is incompatible with normal electron tunneling.

## Results and Discussion

As depicted in Fig. 1b, the single sandwich-type meta-junctions were fabricated by transferring a freshly prepared monolayered Au@SiO_2_ nanomembrane gently, via floating from the “soft” air−water interface onto ITO electrodes, as described previously^21^. To prepare n-layered junctions, this process was repeated n times. The nanomembrane is made of close-packed Au@SiO_2_ core-shell NPs with controllable and homogenous silica thickness ranging from ~5.0 ± 1.1 nm to 14.5 ± 1.7 nm, i.e., a total SiO_2_ thickness from 10–29 nm (Supplementary Fig. S1). Typically, 3 mL colloidal Au@SiO_2_ NPs with silica shell of varied thickness were prepared^22^ (see Supplementary Fig. S2 for UV-Vis characterizations) and poured into a plastic container; 470 μL hexane was then added to the solution to form a liquid/liquid interface, and 3.7 mL methanol was poured into the mixture rapidly to capture the NPs at the hexane/water interface. After hexane evaporation, the NPs simultaneously self-assembled into a golden yellow monolayer membrane over a large area (~cm2-level) at the water/hexane interface and can be seen with the naked eyes (Supplementary Fig. S3). Then the monolayer nanomembrane was transferred onto an ITO electrode. In order to ensure the compactness of the membrane and robustness of the sandwich-type nanocircuits for reliable I-V measurements, we assembled three layers of the nanomembranes by repeating the operation above. Finally, the “lift-off, float-on” (LOFO) technique^20^ was used as a “soft”, nondestructive way to deposit gold pads (~40 nm thick, 0.5 mm in diameter) on the nanomembranes to form planar meta-junctions.

**Figure 2:**
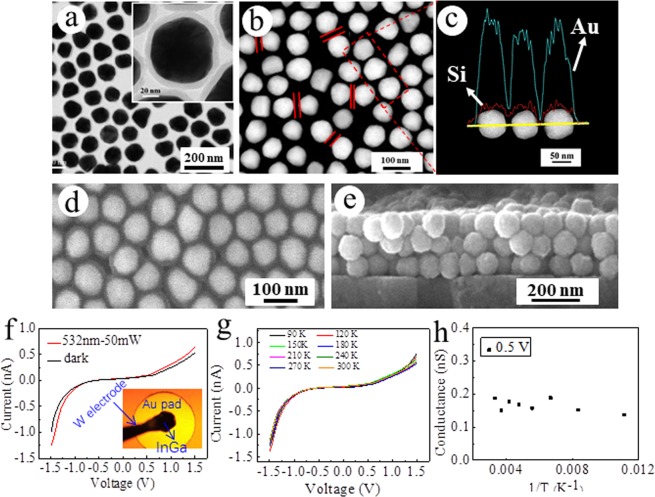
Characterizations and I-V measurements of the as-prepared 75 nm Au@8.1 nm SiO_2_ nanomembrane and tri-layered planar junctions. (**a**) TEM image of monolayered nanomembrane of Au@SiO_2_. (**b**,**c**) HAADF-STEM image and corresponding elemental distribution along the scan line in part (**b**). (**d**) Planar view SEM image of monolayer nanomembrane. (**e**) Cross-section SEM image of tri-layer nanomembrane. (**f**) I–V curves of the tri-layer Au@SiO_2_ meta-junction, recorded in the dark and with 532 nm laser (50 mW) illumination, at room temperature. Inset: Microphotograph of the measured sandwich-type junction. (**g**) I–V curves of a tri-layer Au@SiO_2_ meta-junction measured at temperatures between 90K-300K. (**h**) Conductance of the tri-layer Au@SiO_2_ meta-junction, measured at temperatures between 90K-300K in the dark at 0.5 V.

As seen from TEM image and line-scan energy dispersive X-ray spectroscopy (EDS) analysis (Fig. 2a–c), the typical monolayer nanomembrane made of 75 nm diameter AuNP with an 8.1 ± 1.3 nm silica shell has AuNPs “jammed” in close contact, but nearly all are well-separated by a transparent gap, which, with a width of ~15.4 ± 2.7 nm (Fig. 2b,c) fits to twice the SiO_2_ shell width. No “fatal” metal (Au) junction or filament formation between adjacent AuNPs was observed by both TEM observation and HAADF-STEM image and corresponding elemental distribution analysis between individual NPs (Fig. 2c), which indicates the stability and shell-isolated nature of the AuNPs. The optical properties of the nanomembrane were then characterized by a microscopy-based selected-area bright field spectrometer. The plasmon band of the nanomembrane red-shifted from ~535 nm for free AuNPs to 560 nm (Supplementary Figs. S2 and S4), due to electromagnetic coupling between the NPs. Figure 2d,e and Supplementary Fig. S5 show typical scanning electron microscope (SEM) top-/side-view images of the nanomembrane and the resulting sandwich-type meta-junction (with top Au pad electrode). The SEM images show that the AuNPs in the nanomembrane are all coated with uniform silica shells (*cf*. Fig. 2d and Supplementary Fig. S5b for wide scope SEM images) and that the nanomembrane, as well as the Au contact on top of it remain intact after the floating transfer to the ITO electrodes (see SEM images in Supplementary Fig. S5), which guarantees the mechanical stiffness of the junction and hence stability of I-V measurement.

I–V measurements of the resulting meta-junctions were carried out at room temperature. For each sample, several small, 0.5-mm-diameter Au pads were deposited on the nanomembranes by the LOFO technique. The circuit was completed by gently placing one tungsten electrode on the ITO electrode and another on the Au pad (with an InGa drop on it to eliminate mechanical (pressure) damage to the film, as shown in the inset of Fig. 2f). Figure 2f shows typical I-V curves of a trilayer 75 nm Au@8.1 nm SiO_2_ nanomembrane-based meta-junction recorded in the dark and when illuminated with 532 nm laser (50 mW). Interestingly, over a ±1.5 V bias range, the I-V curves showed observable current transport with nonlinear characteristic, despite the up to 8.1 nm insulating silica shell. The exponential increase of the current with increasing bias is consistent with tunneling of electrons through the trilayer meta-junction. To test this conjecture, temperature dependence of electronic conductivity was measured. As seen in Fig. 2g,h and Supplementary Fig. S6, the conductance is roughly constant over the temperature range tested, again consistent with electron tunneling. Although the conductance in 1.5 V shows little difference over different temperature, the fluctuation is irregular and the (quasi-) ballistic transport can not be the dominant mechanism as ballistic transport has significant relationship with temperature and impurities in materials; Meanwhile, the dimension of the nanoelectronic junctions are much larger (260~nm for trilayer-based nano-junction) than the mean free path of conductive electron in Au (~37.5 nm), which further rule out the leading role of the (quasi-) ballistic transport regime.

To test if electromigration of Au occurs under bias, which will damage the device during electrical measurements, we performed repeat I-V experiments on a junction fabricated with a nanomembrane of a 75 nm Au@8.1 nm SiO_2_ trilayer for 9 cycles, and found that the junction current was quite stable and no sharp increase was detected, which rules out Au electromigration (Supplementary Fig. S7a). This was further confirmed by direct cross-section-view SEM image of the junction (*cf*. Supplementary Fig. S5e,f) and microscope-based selected area bright field spectra (Supplementary Fig. S7b) of the nanomembrane. These results show no evidence of Au filament formation (SEM) or spectrum change of the LSPR band (which is very sensitive to changes in particle size and shape) before and after the I-V test.

To test if the silica shells indeed provide a continuous conformal coat that covers all of the AuNP surface, as shown in the SEM cross-section images (Supplementary Fig. S5e,f) and that the observed current is not due to (partial) short circuit or filament formation, we performed the following test:

A triple membrane layer (75 nm Au@8.1 nm SiO_2_) was deposited on a conducting substrate (ITO), which was electrically connected to a W electrode. A drop of liquid Hg was put on the membrane and a W electrode was inserted into the Hg drop. A voltage was applied between the ITO substrate and the electrode, from −0.5 to 0.5 V, and the resulting current was measured. This was repeated a few times over the course of several minutes. We also applied a fixed 0.1 V voltage and followed the current that flowed as a function of time (Supplementary Fig. S8a,b). The absence of any sign of short-circuit proves that the SiO_2_ shells protect the AuNPs hermetically as otherwise shorts would form, because Hg will immediately amalgamate with Au. Such amalgamation proceeds also without direct physical liquid/solid contact, as the high vapour pressure of Hg leads to amalgamation through any holes/cracks in the SiO_2_ shells. The resulting I-V curves are asymmetric, due to the asymmetry of these junctions. At the fixed voltage the current across the 75 nm Au@8.1 nm SiO_2_ nanomembranes-based meta-junctions remains quite stable during sequential I-V tests over a period of 20 min (Supplementary Fig. S8a) and during 10 min of −100 mV constant applied bias (Supplementary Fig. S8b). These results indicate that the silica shells cover the AuNPs well, so that we can rule out

-a- direct Au-Au contact

-b- incomplete coverage of the SiO_2_ shells.

Normally the quantum mechanical tunneling efficiency over insulating gaps ≥2.5 nm is too small to yield measurable currents, as the tunneling probability (T) is an exponential function of the barrier length, L. T for a single 16.2 nm silica gap $\sim 3\times {10}^{-94}\,{\rm{as}}\,{\rm{T}} \sim {e}^{-\beta L}$, where β is the tunneling decay parameter in units of (length)^−1^ and we used value of 13.3/Å for SiO_2_^2^. The current at rather low applied voltage might be a reflection of a new electron transport regime, via plasmonic coupling^21^ between individual shell-insulated AuNPs.

To confirm this assumption, the underlying mechanism was primarily probed by measuring the dependence of the junction current on silica shell thickness and on AuNP size. As shown in Supplementary Figs. 3a and S9, when the SiO_2_ shell thickness is increased from 5.0 ±1.1 nm to 14.5 ±1.7 nm (*cf*. Supplementary Fig. S10 for size distribution), the junction current drops exponentially from the nA to the pA range, fitting the following equation (for the current in the dark):

$${\rm{I}}-9.18=1.3\times {10}^{5}\exp \,(\,-\,0.83{\rm{L}})$$

where I is the current (pA) and L is the assumed separation distance (width of silica gap, i.e., 2 × width of SiO_2_ shell) between NPs (Å). 9.18 may be the leakage current (in pA). This equation is similar to that for tunneling of charge

${\rm{I}}=\mathrm{Bexp}(-{\rm{\beta }}L),$where B is a pre-exponential factor and β is related to the energy barrier ϕ^23^. In our experiment, we find a β value of 8.3 nm^−1^, lower than that for tunneling across an insulator, 13.3 nm^−1^ for silica^24^. As such value is obtained from measurements in the dark (without possible effects of optically-exited plasmons), we invoke the contribution of “dark” plasmons to electron transport^16^. Plasmonic dark modes are pure near-field modes that can arise from the plasmon hybridization in a set of interacting nanoparticles. And this mode does not couple to light and are difficult to measure with light^25–27^. The dark mode plasmonic effect occurs in the dark, and upon light irradiation, light-excited plasmon modes will couple to the dark mode and enhances the plasmonic effect, which result in an increase of the current. As electrically-excited surface plasmons (dark plasmons) can act in ways similar to those of optically-exited ones^28^, such SP excitation can change the energy of the system, decrease the energy barrier for electron transfer and, thus, improve the tunneling efficiency^29,30^. However, possibly due to the electrically driven plasmonic effect at relatively high bias (with high excited energy, eg. at 1–1.5 V) may be more effective than optically exited one, the percentage change in current for 14.5 nm shell thickness upon exposing light is much tiny than that of the 5 nm and 10.7 nm shell thicknesses. Moreover, if we reduce the size of AuNPs from ~75 ± 2.3 nm to ~32 ± 1.1 nm (keep the SiO_2_ shell at a constant 7–8 nm), a strong decrease in junction current is seen, with typical currents through the ~15-nm-thick insulator layers dropping from ~32.4 to 3.1 pA at 0.5 V applied bias, as shown inFig. 3b and Supplementary Fig. S11 (Detailed characterizations of the nanomembranes used in these experiments are shown in Supplementary Figs. S12 and S13). This result can be construed as support for the involvement of surface plasmon (SP) coupling because with the decrease of the size of plasmonic NPs (typically within the tens of nanometers), the electromagnetic field intensity decreases gradually^31,32^. For Au@SiO_2_ NPs with constant silica shell thickness, AuNPs with smaller diameter will produce weaker electromagnetic field intensity, so the plasmon coupling in the close-packed NPs decrease. Thus, the efficient propagation length of the SP coupling reduces with the decrease of NP size and this length should limit the insulator thickness^33^.

**Figure 3:**
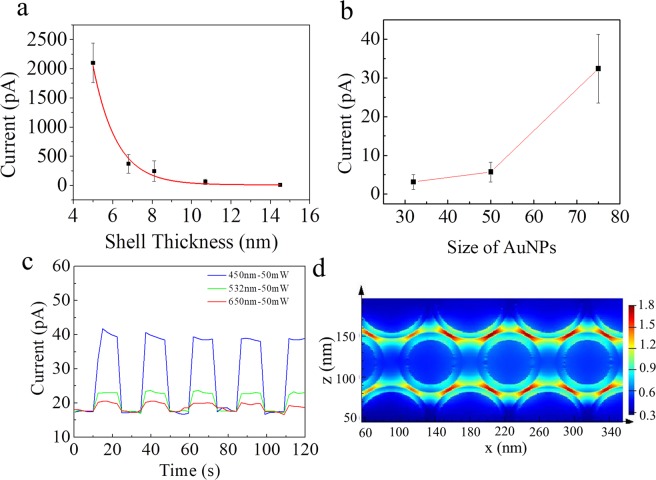
Size-and wavelength-dependent I-V measurements and FDTD simulation of the tri-layer Au@SiO_2_ meta-junctions. (**a**) Influence of silica shell width on the current in the dark (keeping the size of AuNPs at 75 nm). (**b**) Influence of AuNP size on the junction current at 0.5 V in the dark (keeping the silica shell width as ~7.5 nm). (**c**) Photocurrent responses for three different photon excitation energies, at room temperature. (**d**) FDTD simulation of the tri-layer Au@SiO_2_ nanomembrane-based sandwich-type junction.

The plasmonic nature of the trilayer meta-junctions was further manifested by i*n situ* dark-field scattering imaging. As seen from Supplementary Fig. S14, the trilayer Au@SiO_2_ nanomembrane is plasmonically active and the property persists after the successive I-V measurements. No current flows through junctions of nanomembranes using NPs of non-plasmonic SiO_2_ of sizes similar to the AuNP-based ones, as building blocks (detailed characterizations see Supplementary Figs S15). Such junctions showed only sub-pA noise current responses (Supplementary Figs S16), which we ascribe to the lack of plasmonic effect with those NPs, as shown in the microscopy-based selected area bright-field microphotograph (Supplementary Fig. S17a) and dark-field scattering images (Supplementary Fig. S17b). In addition, the optical band gap of pure SiO_2_ shells (etching the 75 nm Au core with aqua regia) was also determined by using the Kubelka-Munk (K-M) function^34^. From the results presented in Fig. S18, the band gap (E_g_) energy of SiO_2_ shell is roughly to be 5.57 eV, which strongly confirmed that the SiO_2_ shell of Au@SiO_2_NPs prepared is insulator, and the I-V characteristics obtained with AuNPs are attributed to their plasmonic properties.

As shown in Fig. 2f, upon irradiation with 532 nm laser (50 mW), the junction current increases from ~0.55 nA to ~0.65 nA at 1.5 V with silica shell thickness of 8.1 nm. Figs. S9 and S19 show similar I-V responses for other junctions. Although the currents vary slightly from junction to junction, the I-V measurements are quite reproducible with respect to current magnitude and all yield similar photoeffects, which indicate the robustness and stability of our meta-junctions. Furthermore, we also observe that the shorter the wavelength, the higher the photocurrent, as shown in Fig. 3c. Thus, it appears that photocurrent enhancement is correlated with the strength of plasmon-induced d → sp interband transitions in AuNPs, which increase in their absorption cross-section at shorter wavelengths^35^. In the blue-visible region, higher energy electrons were excited and participated in the electron transfer, which finally increase the tunneling probability^36^. In addition, complex plasmonic nanoparticle coupling among 2D close-packed nanomembrane may also result in enhanced interaction between nanoparticles and optical radiation, which ultimately leads to the increase of tunneling current with decreasing the wavelength of light^37–39^.

We performed finite difference time domain (FDTD) simulations (based on FDTD Solutions software, Lumerical Inc.) to provide an insight into the electromagnetic field of the particles. As shown in Fig. 3d, there is an enhanced electromagnetic field between adjacent Au@SiO_2_ NPs. Such strong surface plasmon (SP) coupling and excited high-energy electrons are thought to facilitate ultra-long-range electron transfer between adjacent AuNPs^23^. According to the semi-empirical formula reported previously^40,41^, the temperature increase on the nanoparticles’ surface can be estimated to be ~1.3 × 10^−5^ °C ≪ 1 °C in our system (Supplementary S1), i.e., any plasmonically induced local thermal effect on junction current is negligible.

**Figure 4:**
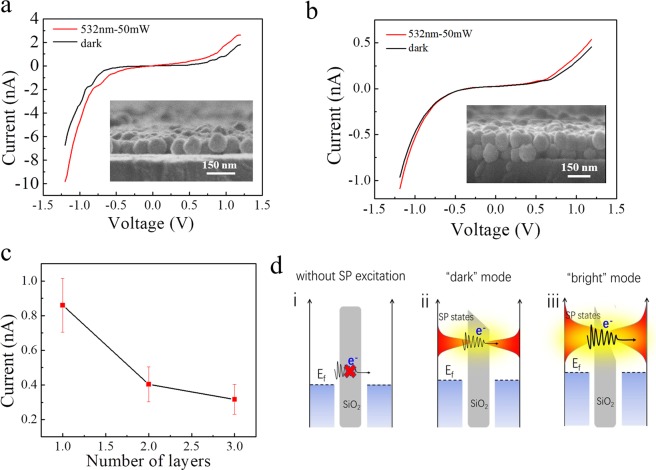
Layer number-dependent I-V measurements and electron tunneling mechanism of the tri-layer 75 nm Au@8.1 nm SiO_2_ nanomembrane-based meta-junction at room temperature. (**a**,**b**) I-V curves of monolayer and bi-layer 75 nm Au@8.1 ± 1.3 nm SiO_2_ meta-junctions. Insets: Cross-section SEM images of the nanomembranes. (**c**) Effect of number of layers on the junction current, recorded in the dark. (**d**) Schematic representations of electron tunnel mechanisms for accepted quantum tunneling, and the proposed P-transport in dark/bright modes.

We further explored the influence of the number of layers of nanomembranes on electron transport systematically. As shown in Fig. 4a–c, although there is a gradual decrease in current with the increase of the number of layers in the nanomembrane, the as-prepared few-layered meta-junctions show an unconventional current decay behavior with obvious nonlinear characteristic over a ±1.2 V bias range (Fig. 4a,b), which may be explained by the suggested P-transport regime. As shown in Fig. 4c, unlike classic tunneling whose tunneling current decreases exponentially with increasing the layer number, the current here does not depend exponentially on the layer number, which is an indicative of unique plasmonic and electronic properties of the 2D Au@SiO_2_ nanomembrane^41^. With the increase of the layer number, the plasmonic coupling in and between layers is intensified and will play an important role for electron transport. Due to the intensive plasmonic coupling electromagnetic effect of the multilayers, the charges may become more delocalized and the overlap of wave functions increases^42^, which will result in the tunneling current increase and does not follow the exponential decay relationship with the increased number of layers. In addition, the conductance behavior is closely related to the distribution and volume fraction of nanoparticles^36^. With the increase of the layer number, the distribution and volume fraction of nanoparticles become different, which may also an important reason of the current deviation from an exponential decrease. To figure out the whole ET scene and for better comparison, current flow through a single 75 nm Au@8.1 nm Silica NP, was estimated to be ~0.04 fA at 1 V as derived experimentally from Fig. 4a. This is much higher (~10^76^ enhancement) than that calculated by accepted electron tunneling (1 × 10^−93^ *A*) with two sequential tunneling steps across two 8.1 nm silica gaps. (see S2 in supporting information for the calculation).

On the basis of the above results, a possible mechanism is suggested in Fig. 4d. In the absence of a plasmon field, the electrons hardly tunnel through the silica shell, due to the large tunneling barrier of insulating SiO_2_ (i). Due to the proximity of strong electromagnetic fields of AuNPs, once a voltage is applied, the electrically-induced dipolar polarization of (dark) plasmon-electrons (p-electrons) will generate plasmon oscillation/coupling and delocalization of the p-electrons, resulting in energetic p-electrons tunneling through the energy barrier of the adjacent Au@SiO_2_ NPs (ii)^29^. Upon illumination with light, the “light” mode plasmons are activated, accelerating (hot) p-electrons to higher energy and decreasing further the energy barrier, which increases the probability of electron tunneling and thus enhances the current, correspondingly (iii). To get the scenario of the whole story, further research using scanning probe microscopy based approaches is needed, although this is a challenge (due to the detection limitation of small currents), it can help really probe its electrical properties on the nanoscale to infer more fundamental insights.

In summary, by constructing few-layered Au@SiO_2_ nanomembranes-based meta-junctions, we observe highly efficient electronic transport over a remarkable distance, which we interpret as (dark, electrically-induced) plasmon-enhanced transport. Remarkable temperature-independent long-range electron transport was observed across the AuNPs with insulating gaps up to 29 nm/NP layer, which cannot be explained by “conventional” electron tunneling. The crucial role of the LSPR coupling on P-transport was evidenced by photocurrent enhancement and FDTD simulation of the electric field distribution. This finding reshapes our thinking on electron transport on the nanoscale and will benefit the design of novel plasmonic nanocircuits and functional devices for various applications.

## Methods

### Materials

Sodium citrate (Na_3_CA), sodium silicate (Na_2_SiO_3_), 3-aminopropyltrimethoxysilane (APTMS) were purchased from Sigma-Aldrich. HAuCl_4_·4H_2_O, methanol, n-hexane were purchased from Beijing Chemical Factory (Beijing, China). ITO-coated glass was purchased from Zhuhai Kaivo Optoelectric Technology Co., Ltd. All of the chemicals were used without further purification. Water used throughout all these experiments was purified with a Millipore system (18.2 MΩ· cm).

### Characterization

The morphologies of the Au@SiO_2_ NPs were characterized with a JEM-2100F transmission electron microscope (TEM) and an XL30 ESEM scanning electron microscope (SEM). The UV−vis absorption spectra were obtained, using a UV-2600 spectrophotometer (Shimadzu, Japan). The cyclic voltammetry curves of Au and pinhole-free Au@SiO_2_ NPs were measured with a three-electrode setup, using an electrochemical workstation (CHI832C). In the three-electrode setup, the working electrode is a glassy-carbon electrode, decorated with AuNPs or with pf-Au@SiO_2_ NPs (prepared by dropping 6 μL of nanoparticles solution on the electrode and dried at the room temperature), while the reference and counter electrodes are Ag/AgCl electrode and Pt wire, respectively. The potential of the test was from − 0.35 to 1.4 V in 0.1 M H_2_SO_4_ at 20 mV/s. Optical microscopy-based selected area dark-field scattering images and spectra were obtained by using an inverted Leica DMI6000B microscope (Germany), connected with a USB 4000 spectrometer (Ocean Optics, Inc.). Room-temperature I-V characteristics of the junctions were obtained at room temperature by using a Keithley 2636B source meter. Lasers with different wavelengths (450 nm, 532 nm, 650 nm) were used for illumination.

### Synthesis of Au@SiO_2_ NPs

The AuNPs of different size were prepared by the methods reported previously^43–45^. Au@SiO_2_ nanoparticles with different silica shell widths were synthesized by the method reported by Tian’s group^46^ with some modification. Typically, fresh prepared 1 mM APTMS was drop-wise added to the gold sol and vigorously stirred for 30 min; then 0.54% Na_2_SiO_3_ was added in the sol and heated at 90 °C for 0.5 to 3 h. Pinhole-free Au@SiO_2_ NPs with tunable shell-thickness can be obtained by carefully controlling the reaction time, pH and concentration.

### Fabrication of few-layer nanomembrane-based sandwich-type junctions

ITO glass was cleaned by sonication with water, acetone, ethanol and finally with water for 15 min, respectively. Then, each slide was placed in a mixture of 5:1:1 H_2_O + 30%H_2_O_2_ + 25%NH_3_ and heated for 40 min at 80 °C. The monolayer Au@SiO_2_ nanomembranes were prepared by the method of liquid/liquid interface self-assembly according to previous research^47^. Then, the nanomembranes were transferred carefully from the “soft” air−water interface onto the ITO electrodes as depicted in our method reported elsewhere^21^. Several layers of nanomembranes were fabricated by repeating the operation above. To form planar sandwiched junctions, we used the “lift-off, float-on” (LOFO) technique as a “soft,” nondestructive way to deposit gold pads (~40 nm thick, 0.5 mm in diameter) on the nanomembranes^20,48^. The devices were thoroughly air-dried before the electrical measurements.

### Characterization of electrical properties

The circuit was completed by placing a W electrode on the Au electrode gently (an InGa drop on the Au minimizes mechanical damage to the film) and another on the ITO glass with a micromanipulator. The quality of as-deposited Au pad was checked under the optical microscope and only those structures where we succeeded in depositing smooth continuous Au electrodes, without wrinkles or visible holes, were used for I-V measurements. All the measurements were performed at ambient conditions. Lasers emitting at λ = 450, 532, 650 nm were used for plasmon excitation of the NPs assemblies. In order to reduce radiative heating effects, the laser beam was focused on nanomembranes via an optical fiber instead of irradiating the membranes directly.

### Electromagnetic field simulation

FDTD simulations were run using the commercially available software developed by Lumerical Inc. The simulation cell of the 2D plasmonic nanomembrane was constructed to the Au@SiO_2_ nanoparticle array and the corresponding electromagnetic field enhancement spectrum was matched to that of the Au@SiO_2_ nanoparticle arrays to ensure consistency. The wavelength of the irradiation was set at 532 nm.

## supplementary material

Supplementary information
