# Supplementary material for: Unprecedented efficient electron transport across Au nanoparticles with up to 25-nm insulating SiO2-shells
Source: Sci Rep. 2019 Dec 4;9:18336. doi: 10.1038/s41598-019-54835-2 (PMC6892908; doi:10.1038/s41598-019-54835-2)
Supplement: Supplementary file 1 — Supplementary information [file 41598_2019_54835_MOESM1_ESM.pdf]

Supporting Information

Unprecedented efficient electron transport across Au nanoparticles with up to 25-nm insulating SiO_2-shells

Chuanping Li[1,2], Chen Xu[1,3], David Cahen[4] & Yongdong Jin[1,2,3,*]

[1]State Key Laboratory of Electroanalytical Chemistry, Changchun Institute of Applied Chemistry, Chinese Academy of Sciences, 5625 Renmin Street, Changchun 130022, P. R. China.

[2]University of Chinese Academy of Sciences, Beijing 100049, P. R. China.

[3]University of Science and Technology of China, Hefei, Anhui, 230029, P. R. China.

[4]Department of Materials and Interfaces, Weizmann Institute of Science, Rehovot 76100, Israel.

Correspondence and requests for materials should be addressed to Y.D.J. (email: ydjin@ciac.ac.cn).

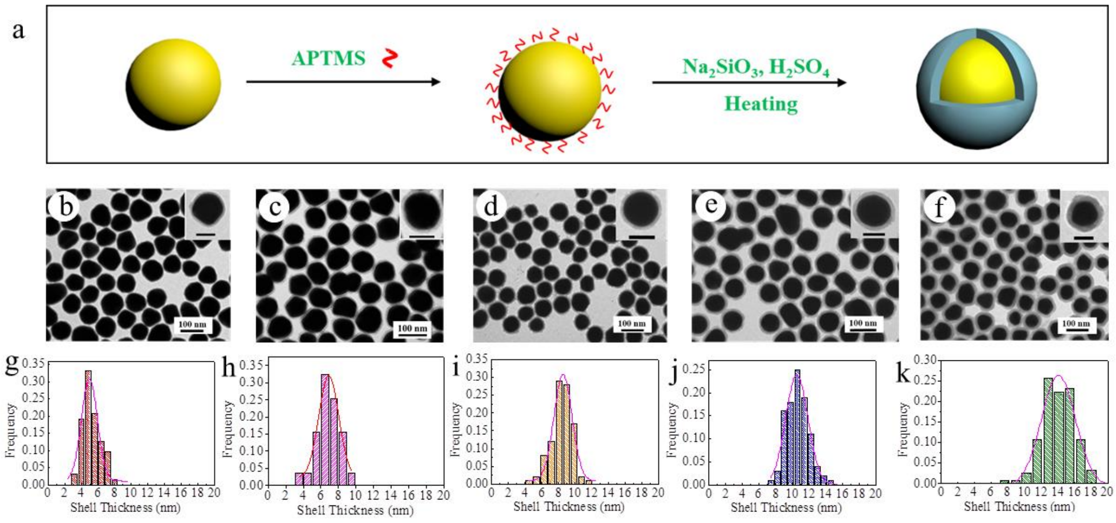

Figure S1| Preparation and size characterization of Au@SiO₂ NPs. a, Schematics of the preparation of Au@SiO₂ NPs and TEM images of Au@SiO₂ NPs with 75±2.3 nm cores and varied silica shell thickness of **(b, g)** 5.0±1.1 nm, **(c, h)** 6.8±1.5 nm, **(d, i)** 8.1±1.3 nm, **(e, j)** 10.7±1.4 nm and **(f, k)** 14.5±1.7 nm.

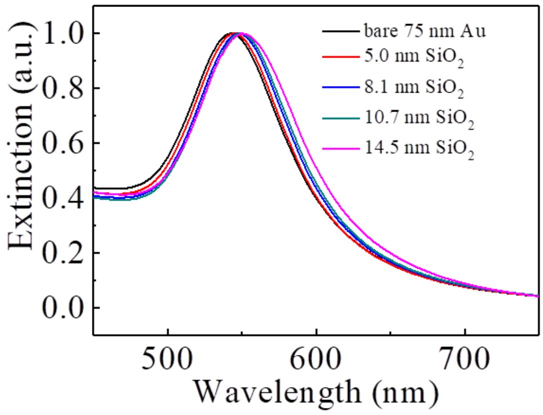

Figure S2| UV-Vis Characterizations of the as-prepared Au@SiO₂ NPs.

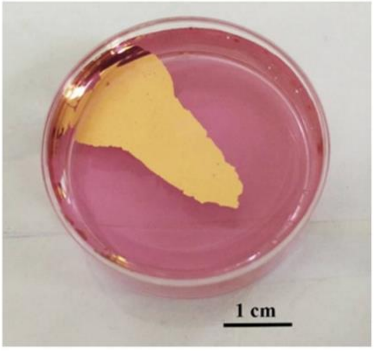

Figure S3| Photograph of monolayer 75 nm Au@ 8.1 nm SiO_2 nanomembrane, assembled at the water/air interface.

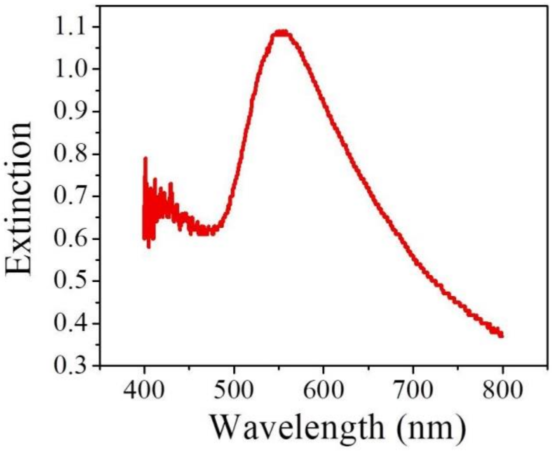

Figure S4| Microscopy-based selected area bright field spectra of the monolayer 75 nm Au@8.1 nm SiO_2 nanomembrane.

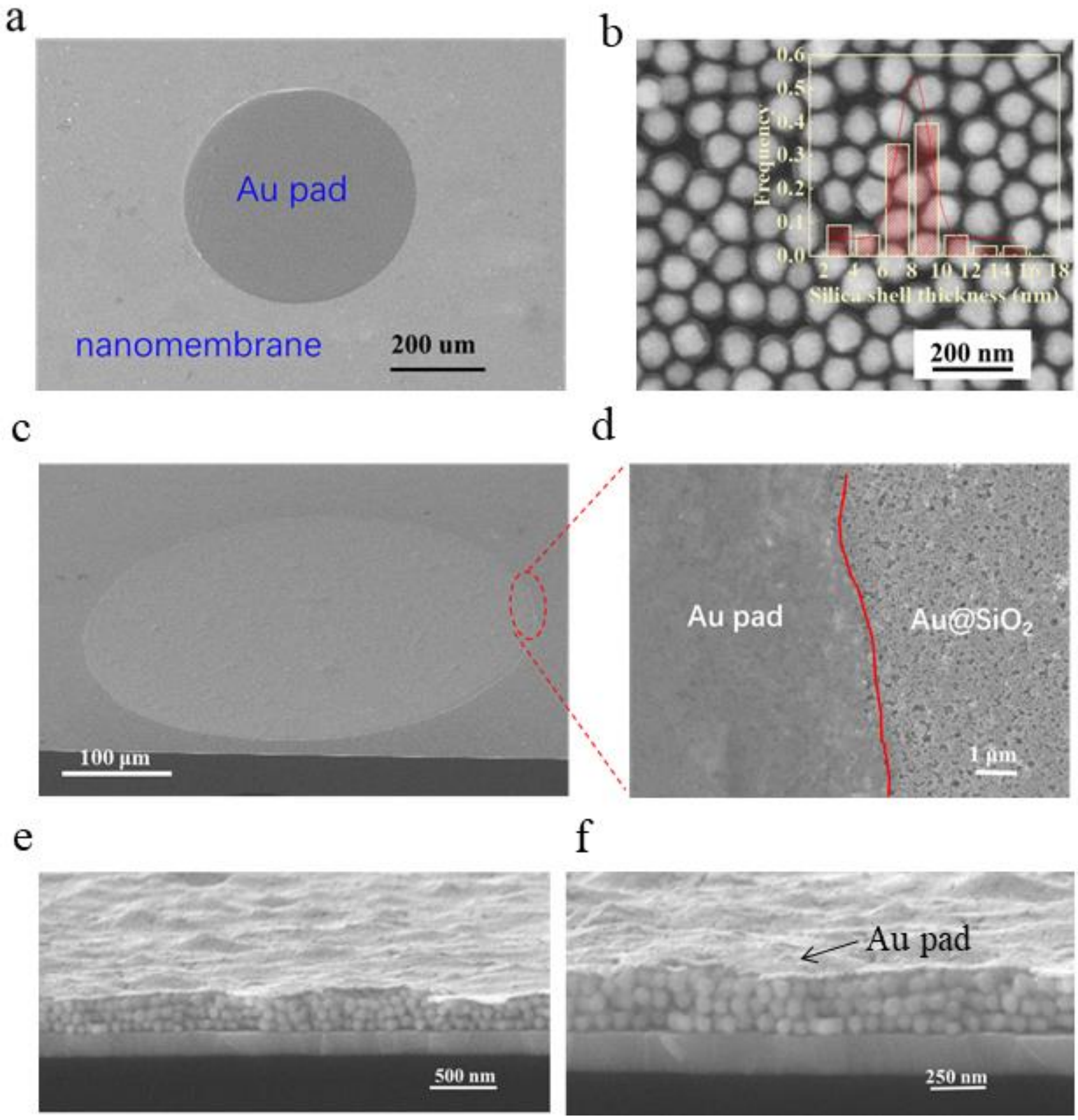

Figure S5| SEM characterizations of the sandwich-type junction. a, a top view of a Au/trimembrane/ITO junction. **b,** A wide scope field SEM image of the monolayer 75 nm Au@8.0 nm SiO_2 nanomembrane, showing (*inset*) a uniform silica shell coating of 8.0±2.2 nm. **c,** SEM image of a Au/trimembrane/ITO junction, viewed from a 60-degree angle. **d,** Enlarged SEM image of the border between the Au pad and the nanomembrane. Note: the nanomembrane and Au pad remain intact after the floating transfer to the ITO electrodes. (**e-f**) Cross section SEM images of a Au/trimembrane/ITO junction after the I-V experiment, which indicates the integrity of the trimembrane and Au pad.

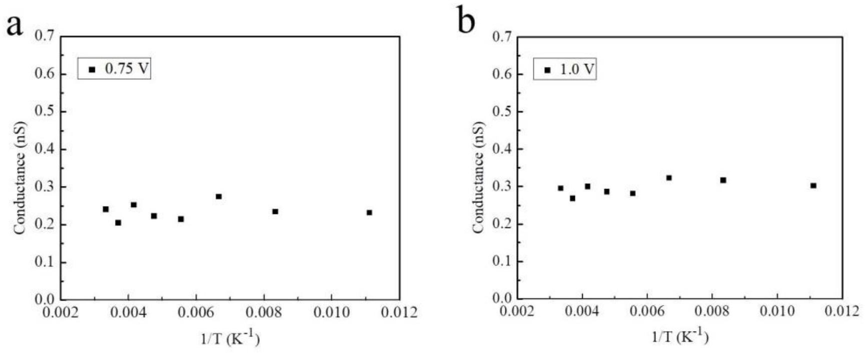

Figure S6| Temperature-independent conductance of the tri-layer meta-junction. Conductance of a 75 nm Au@8.1 nm SiO_2 tri-layer meta-junction, measured between 90K-300K in the dark at **a,** 0.75 V and **b,** 1.0 V.

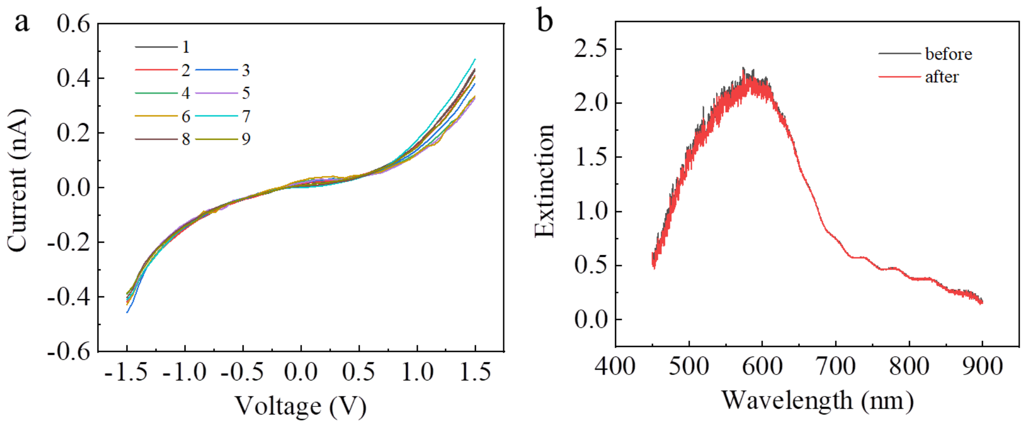

Figure S7| Repetitive experiments of the tri-layer nanomembrane-based meta-junction. a, I-V curves of a 75 nm Au@8.1 nm SiO_2 tri-layer nanomembrane-based meta-junction, measured in the dark for 9 successive cycles. **b,** Microscopy-based selected area bright field spectra of a tri-layer 75 nm Au@8.1 nm SiO_2 nanomembrane before and after the 9 cycles of I-V tests.

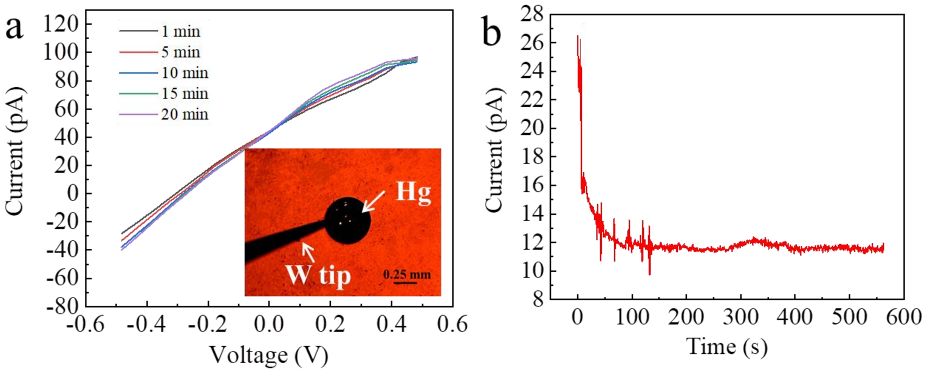

Figure S8| Robustness tests of the tri-layer nanomembrane-based meta-junction. a, I-V curves of the 75 nm Au@8.1 nm SiO_2 nanomembrane-based meta-junction, measured by putting a small Hg drop on the nanomembrane as the top electrode and tested every 5 minutes. *Inset*: Optical microphotograph of the measured meta-junction. **b,** I-t curve of the device at -100 mV applied bias during a 10 min test. The sudden decrease in current at the start of the experiment may be due to charging effects at the Hg electrode interface, or possible contamination of the Hg with time under ambient testing conditions.

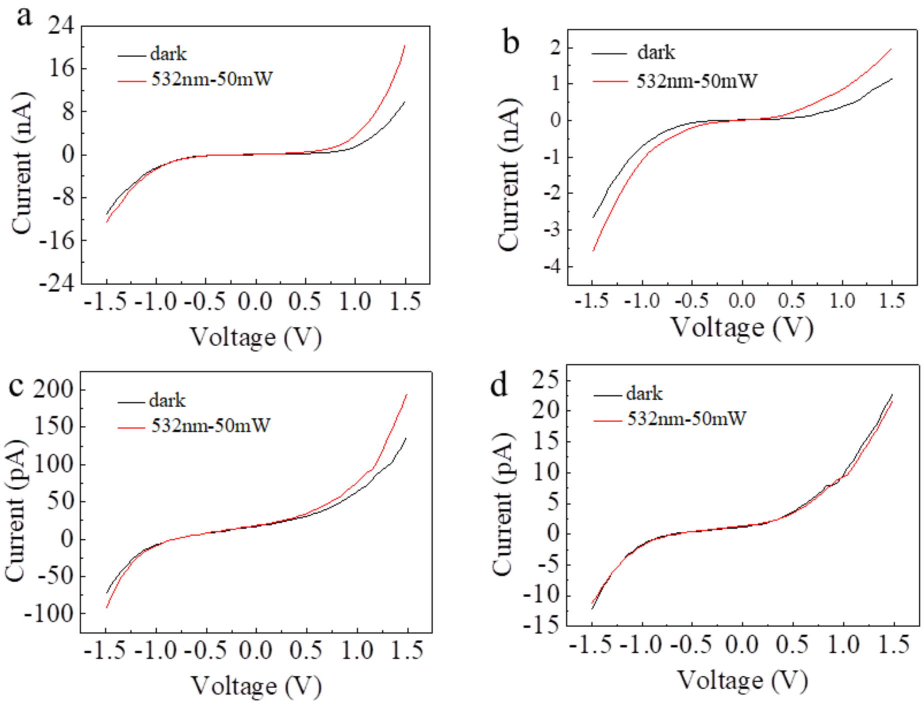

Figure S9| Shell thickness dependent (photo)current of the meta-junctions. I-V curves of the tri-layer 75 nm Au@SiO_2 nanomembrane-based meta-junctions measured in the dark and under illumination of 532 nm laser (50mW) with different silica shell widths of **a,** 5.0 nm, **b,** 6.8 nm and **c,** 10.7 nm, **d,** 14.5 nm.

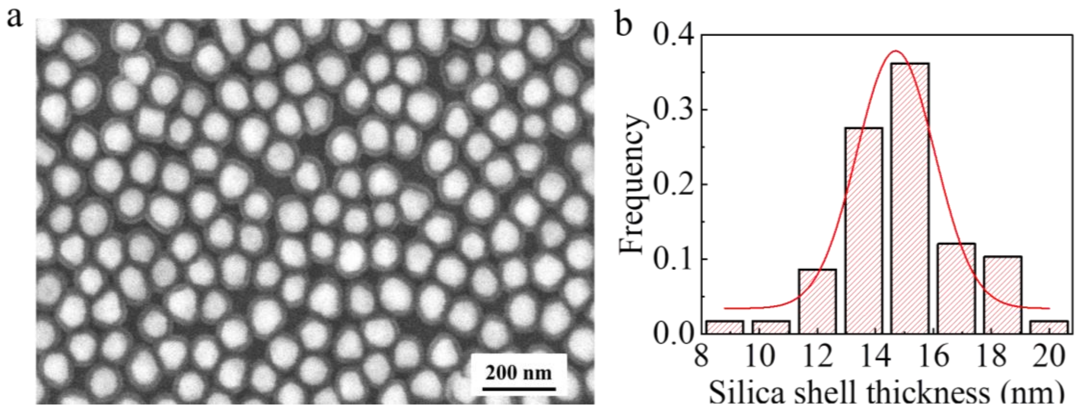

Figure S10| SEM image of a typical monolayer 75 nm Au@14.2 nm SiO_2 nanomembrane. a, SEM image of a typical monolayer 75 nm Au@14.2 nm SiO_2 nanomembrane. **b,** Distribution of silica shell thicknesses of the nanomembrane, showing a uniform silica shell coating of 14.2±2.9 nm.

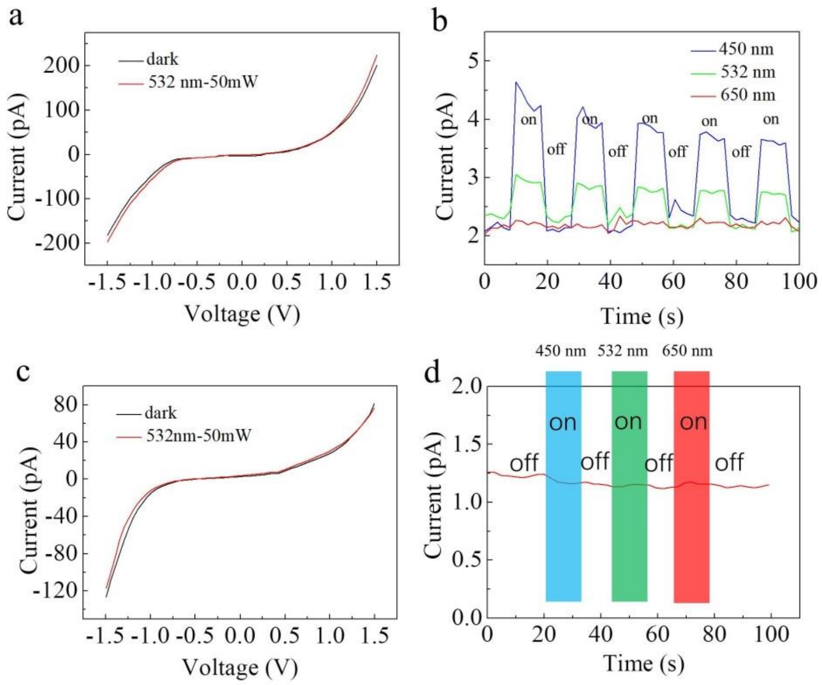

Figure S11| AuNPs-size dependent (photo)current of the meta-junctions. a, I-V curves of a tri-layered 50 nm Au@7.8 nm SiO_2 nanomembrane-based meta-junction measured in the dark and under the illumination of 532 nm laser (50mW). **b,** corresponding wavelength-dependent photocurrent response and stability at 0.5 V with the same light intensity of 50 mW. **c,** I-V curves of the tri-layer 32 nm Au@7.6 nm SiO_2 nanomembrane-based meta-junction in the dark and under 532 nm, 50 mW laser illumination; **d,** corresponding wavelength-dependent photocurrent response at 0.5 V with the same light intensity of 50 mW.

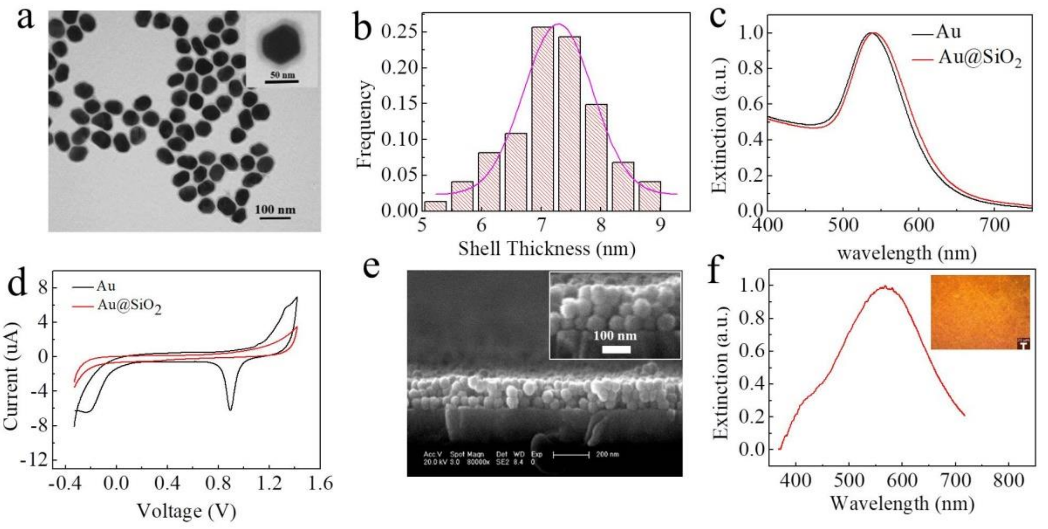

Figure S12| Characterizations of a tri-layered 50 nm Au@7.8 nm SiO_2 nanomembrane-based meta-junction. a, TEM image of the 50±3.7 nm Au@7.8±0.8 nm SiO_2 NPs. **b,** Silica shell thickness distribution of 50 nm Au@7.8 nm SiO_2 NPs. **c,d** UV-vis spectra and cyclic voltammetry (CV) curves of the control 50 nm AuNPs and 50 nm Au@7.8 nm SiO_2 NPs. **e,** Cross-section images of the tri-layered 50 nm Au@7.8 nm SiO_2 nanomembranes. **f,** microscopy-based selected area bright-field extinction spectra of the tri-layered 50 nm Au@7.8 nm SiO_2 nanomembrane. Inset: Dark-field scattering image of the tri-layered 50 nm Au@7.8 nm SiO_2 nanomembrane.

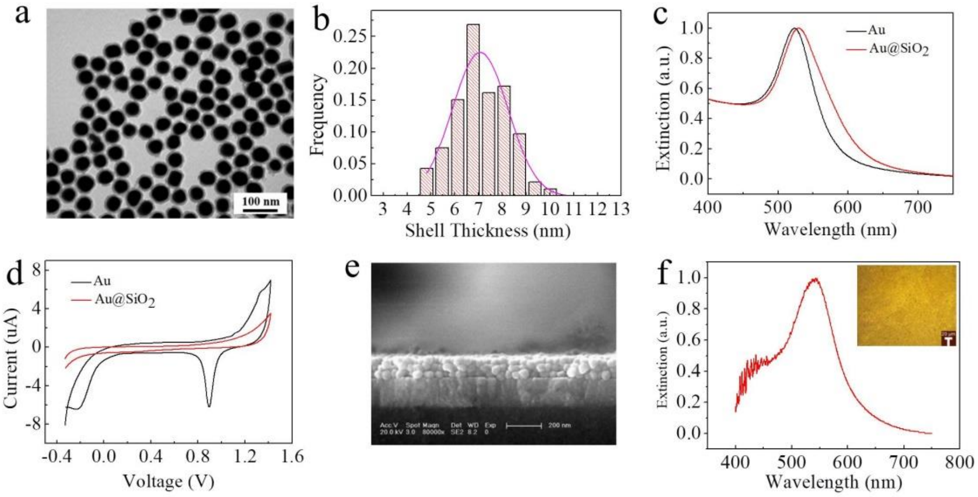

Figure S13| Characterizations of a tri-layered 32 nm Au@7.6 nm SiO_2 nanomembrane-based meta-junction. a, TEM image of the 32$\pm$1.1 nm Au@7.6$\pm$0.9 nm SiO_2 NPs. **b,** Silica shell thickness distribution of the 32 nm Au@7.6 nm SiO_2 NPs. (**c,d**) UV-vis spectra and cyclic voltammograms of the control 32 nm AuNPs and 32 nm Au@7.6 nm SiO_2 NPs. **e,** Cross-section image of the tri-layer 32 nm Au@7.6 nm SiO_2 nanomembrane. **f,** Microscopy-based selected area bright-field extinction spectra of the tri-layer 32 nm Au@7.6 nm SiO_2 nanomembrane. *Inset*: Dark-field scattering image of the tri-layer 32 nm Au@7.6 nm SiO_2 nanomembrane.

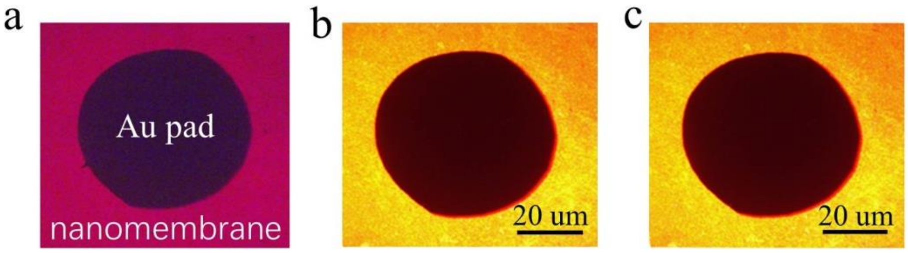

Figure S14| Characterization of plasmonic activity of the sandwich-type meta-junction. a, Microscopy-based selected area bright-field microphotograph and dark-field scattering images of the tri-layer 75 nm Au@8.1 nm SiO_2 nanomembrane-based sandwich-type meta-junction before (**b**) and after (**c**) I-V measurements at room temperature.

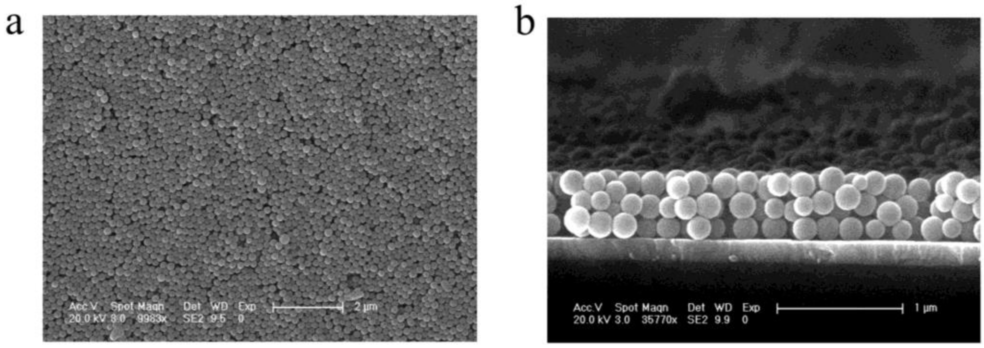

Figure S15| Characterization of the SiO_2-based meta-junction. a, SEM image and **b,** cross-section image of the tri-layer nanomembrane made of the control 80±11nm SiO_2 NPs.

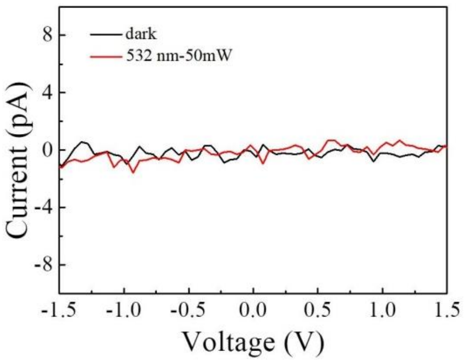

Figure S16| I-V curves of the control tri-layer SiO_2 nanomembrane-based sandwich-type junction at room temperature.

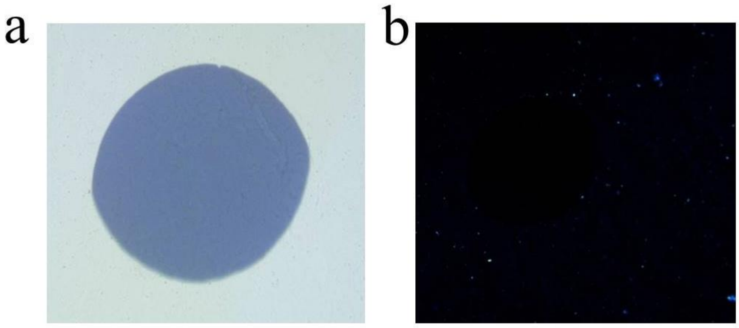

Figure S17| Optical characterization of the tri-layer SiO_2 nanomembrane-based junction. a, Optical microscopy-based selected area bright-field microphotograph and **b,** dark-field scattering images of the control tri-layer SiO_2 nanomembrane-based junction.

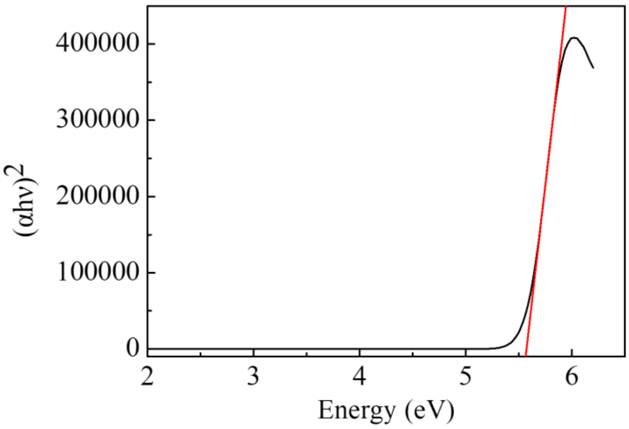

Figure S18| Plots of the transformed Kubelka–Munk function vs. the energy of light for SiO_2 shell.

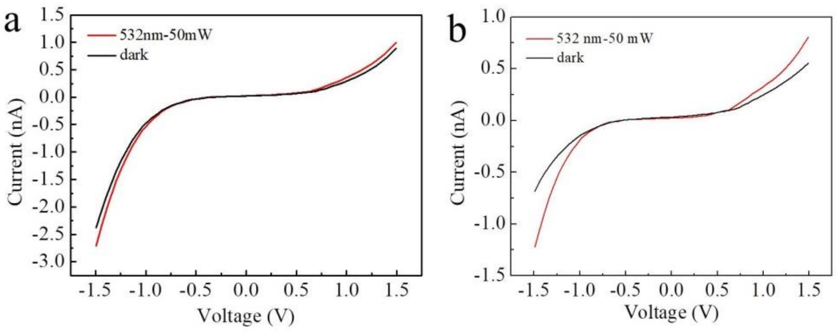

Figure S19| Layer number-dependent I-V measurements at room temperature. I-V curves of another tri-layer 75 nm Au@8.1 nm SiO_2 nanomembrane-based meta-junctions measured in the dark and under 532 nm, 50 mW laser illumination.

S1| Temperature increase (ΔT) estimation:

According to earlier reported research[1], the plasmon-induced temperature increase (ΔT) can be calculated by the following equations:

$$Q_{nano}=C_{abs}\times I$$

$$\Delta T_{nano}=Q_{nano}/(4\pi k_s r_{NP})$$

where Q_{nano} is the quantity of heat produced by the Au@SiO_2 NPs, C_{abs} is the absorption cross-section area of AuNPs (m^2), I is the light intensity impinging on the nanomembranes (W/m^2), r_{NP} is the particle radius of AuNPs, and k_s is the thermal conductivity of the silica ($W\cdot m^{-1}\cdot K^{-1}$).

We will do this calculation with parameters of a real experimental system: I = 110 mW/cm^2, r_{NP} = 37.5 nm, k_s = 0.52 $W\cdot m^{-1}\cdot K^{-1}$ (for silica shell), and C_{abs} = 2827 nm^2. Then the temperature increase ΔT_{nano} is calculated to be ~ $1.27*10^{-5}$ °C, which is negligible, i.e., any photothermal effect on the electron transfer will be negligible.

S2| Comparison of the junction current flow through a single 75 nm Au@8.1 nm SiO_2 nanoparticle, derived from our I-V measurements (for P-tunneling) or calculated from accepted electron tunneling:

(1) Junction current flowing through a single 75 nm Au@8.1 nm SiO_2 NP derived from I-V measurements:

The nanoparticle density is ~120/um^2 (**Figure S5b**) and the area of the Au pad is ~$2\times 10^5 \mu m^2$, which is the contact area. Thus, ~2.4×10^7 nanoparticles can contribute to the current flowing across the junction. From the results of our experiment (0.98 nA at 1 V, **Figure 4a**), the measured current (at 1 V) is ~0.04 fA per nanoparticle (or more, if not all particles are contacted).

(2) The tunneling current that can flow through a single 75 nm Au@8.1 nm SiO_2 NP, can be estimated, using the WKB model[2],

$$j\approx 2G_0 e^{-\beta L} V/a^2 \approx 1\times 10^{-52} A/Å^2$$

where G_0 is the quantum of conductance, 77 uS, β is a length decay factor (in nm^{-1}) and is a function of the energy (13.3 nm^{-1} for SiO_2 at 4 eV); L is the width of the silica shell (8.1 nm). V is the applied bias (we will use 1 V) and a = 5.06 Å, the lattice constant for the conventional unit cell used in SiO_2 band structure calculations.

The contact area S is $\sim 2\times 10^{-3}$cm^2 (~$2\times 10^5 \mu m^2$).

$$I=j*S=2\times 10^{-39}\ A$$

The tunneling probability is $e^{-\beta L}(\sim 1.6\times 10^{-47})$ for a single 8.1 nm silica gap. Furthermore, electrons cross the electrode/AuNPs twice (from one electrode to the AuNP and then from AuNP to another electrode) to get from one electrode to the other one. Thus, the estimated current for the junction is ~3×10^{-86} A.

In other words, based on the accepted electron tunneling model, if we want to observe a measured current (at 1 V) of ~0.04 fA per nanoparticle, the tunneling distance (across SiO_2) can be only ~2.1 nm, which is much shorter than the P-tunneling distance (~16 nm).

Schematic diagram we used to calculate the electron tunneling current across a single nanoparticle by using the WKB model.

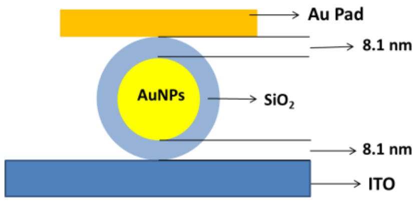

References:

1. Qin, Z. & Bischof, J. C. Thermophysical and biological responses of gold nanoparticle laser heating. *Chem. Soc. Rev.* **41**, 1191-1217 (2012).
2. Tomfohr, J. K. & Sankey, O. F. Simple Estimates of the Electron Transport Properties of Molecules. *Phys. Status Solidi B* **233**, 59-69 (2002).
